# General Medicine and Therapeutics

**Published:** 1893-04

**Authors:** 


### GENERAL MEDICINE AND THERAPEUTICS.

EDITED BY DR. LUTHER B. GRANDY.

### DEPARTMENT OF MEDICINE AT THE WORLD'S FAIR.

The plans for medical service, promulgated by the Medical Director of the Exposition, contemplate the maintenance of a hospital in which the more advanced modern theories will be demonstrated. A striking feature of this will be an effective ambulance service, each ambulance being accompanied by a trained nurse when called for the removal of a patient to the hospital. Here the Board of Lady Managers perceived its opportunity, and at once began the foundation of an elaborate scheme, the establishment and maintenance of a series of movable hospitals or relief stations, at various points on the World's Fair grounds, by means of which the safety and comfort of the public would be greatly served. Patients requiring immediate attention can be taken into one of these relief stations and there receive such prompt, expert care as will often render unnecessary their removal to the hospital. In addition to the opportunity thus afforded for a practical exhibition of woman's skill as a trained nurse, which was the original incentive of the lady managers, this plan also gave scope for comprehensive exhibits. The idea of the lady managers has received the most cordial approval of the Chief of Liberal Arts. Fearing, however, lest this service might be rejected by the directory in the event of its entailing any expense upon the Exposition, those most vitally interested—the great hospitals, the makers of hospital fittings and appliances—readily agreed to establish and maintain these relief stations at their own expense, feeling recompensed by the privileges of exhibition. Large allied interests are represented in the united movement, the list of hospitals including such names as the Bellevue, the Mt.

Sinai, the Hahnemann, the Roosevelt, the New York Hospital—all of New York City—and the great hospitals of Pennsylvania and Massachusetts.—*Med. Fortnightly.*

---

### YELLOW FEVER INOCULATION.

Dr. J. McFadden Gaston, of this city, has lately contributed letters to the *Journal of the American Medical Association* and to the *Medical News* with respect to the results of experimental inoculation for yellow fever in Brazil. The data of his articles are fully corroborated by a report of the recent work of Domingos Freire, published in the *Jornal de Brazil*, January 28th :

"VACCINATION AGAINST YELLOW FEVER.

"Dr. Domingos Freire writes us : 'The total number of inoculations for the prevention of yellow fever during the epidemic of 1891-92 was one thousand. There were 377 foreigners and 623 natives.

"'Nearly the total number were in the most probable condition to contract the disease. The great majority had been in Rio de Janeiro varying from a few days to four years. Then, in calculating the lowest period of receptivity, it is put down as five years' residence in the infected place as the minimum period for acclimatization. The individuals inoculated lived, for the most part, in the streets where the disease had become most widely developed.

"'The mortality among those inoculated was 0.8 per cent. (eight-tenths of 1 per cent.) During the same epidemic there died about four thousand people who had not been inoculated. From 1883 to 1892 there have been 11,881 inoculations to prevent yellow fever, with a mortality of 0.5 per cent. (five-tenths of 1 per cent.)

"'The results of the inoculations show that this prophylactic means has a real efficacy.'"

### THE QUACK QUESTION AS HANDLED BY THE CITY COUNCIL OF MANSFIELD, OHIO.

At the meeting of the council of the city of Mansfield, Ohio, on November 29th, 1892, an ordinance was passed by a two-thirds majority which prevents any quacks or itinerant venders of medicine, "toothpullers" or other impostors practicing their nefarious schemes in that city without first getting a permit from the health officer, who, by the ordinance, is required to be a regular physician. The ordinance also requires these quacks to display a diploma from some respectable college before the health officer can give them the necessary certificate entitling them to a license at all. On the presentation of said certificate to the mayor they can receive a license for which they must pay not less than $25 nor more than $50 a day, and are also subject to a fine of not less than $25 nor more than $50 for each and every offence for the violation of this ordinance.

The law goes into effect immediately after its publication and covers physicians, mid-wives, pharmacists and dentists. If every city council throughout the State of Ohio would follow the example set by the council of Mansfield they would take a grand step in the direction of getting rid of quacks and impostors which infest all our large cities. This plan has been tried in Kentucky, and so far has proved to be of great advantage in getting rid of these leeches, and should be followed by all the States that have no special laws or that cannot get special legislation to remedy this great evil.—*Exchange.*

---

### GUAIACOL IN THE TREATMENT OF TUBERCULOSIS.

Dr. A. Jacobi read a paper before the Climatological Association upon the treatment of pulmonary tuberculosis by guaiacol, which was first recommended in 1880 by Shueller. Guaiacol is an ethereal product of beech wood which is soluble in two hundred parts of water. It is the principal constituent of creosote, which contains it in varying proportions, somewhat over fifty per cent., and is preferable to the latter in that it is better borne by the stomach. It may be given in the form of carbonate, a description of which will be found in the lecture by Dr. Kinnicutt in the *Journal* of May 26, 1892.

Dr. Jacobi gives the remedy almost exclusively in four doses daily after meals and at bedtime in sweetened water or in milk. He begins with four drops for adults, and never increases to more that twenty-eight, and for a child twelve. It is occasionally necessary to discontinue it temporarily. Most of the patients after one to three months looked better and felt better. Some had gained in flesh; the cough became looser and appeared to be more mucous and less purulent. The author had previously obtained such good results from arsenic and digitalis that in at least half of his cases he combined them with the use of guaiacol. Cod liver oil also was given in cold water to patients with fair digestion.—*Boston Med. and Surg. Journal.*

---

### TURPENTINE IN TYPHOID FEVER.

Dr. H. C. Wood (*Therap. Gaz.*) believes that turpentine is of great service in healing intestinal ulcers, which, after the fever, so often cause diarrhœa and intestinal indigestion. Again the remedy is of great service when we have tympanites with dryness of the tongue, developing in the end of the second week of the disease. Wood, believing that turpentine acts locally, that in all cases of typhoid fever ulceration exists, that properly administered the drug is incapable of doing harm, is in the habit of giving towards the close of the second week of typhoid fever turpentine, without looking for special indications, and, as the result of an experience of considerably over a quarter of a century, he believes the practice to be a good one, and that the use of turpentine does distinctly tend to lessen the severity of the local lesions in enteric fever. In closing this brief paper, attention is called to the powers of glycerine in disguising the taste of turpentine; the following formula is given:

℞. Ol. terebinthinæ, f ʒ viii.

Glycerinæ, f ʒ i.

Mucil. acaciæ, f ʒ iss.

Aquæ menthæ piperitæ, q. s. ad f ℥ viii.—

M. Sig.—Tablespoonful every four hours during the day. Shake well.—*Canada Lancet.*

### FOR CONSTIPATION.

The following prescription is recommended :

℞. Ext. cascaræ sagradæ, fl, Glycerinæ, } aa f ʒ i ♏ xx.

Aquæ, qs. ad., f ℥ ij.

M. et Sig.—ʒ i. at bedtime to a young infant.

When the stools are hard and clay-colored, the following has been recommended by Ringer :

℞. Resinæ podophylli, gr. i.
Alcoholis, f ʒ i.

M. et Sig.—One or two drops on sugar t. i. d. to infant one or two months old.—*Lancet-Clinic.*

---

### SUMMER REGIMEN FOR NURSING INFANTS.

DURING the variable weather of summer, when digestive disturbances are so apt to occur in nursing infants, Toussiant advises a decided decrease in the amount of milk taken, and prescribes the following to be taken after each feeding :

℞. Papaine pur., gr. vi.
Acid lactic, gr. xxiv.
Symp. simplicis, f ℥iss.
Tr. vanilla, q. s
Aquæ, f ℥iiiss.

M. S.—A dessertspoonful after each meal.—*Annals of Gynecology.*

